# Behavior and interactions of the plant growth-promoting bacteria *Azospirillum oryzae* NBT506 and *Bacillus velezensis* UTB96 in a co-culture system

**DOI:** 10.1007/s11274-022-03283-8

**Published:** 2022-04-29

**Authors:** Negar Bagheri, Masoud Ahmadzadeh, Pierre Mariotte, Gholamreza Salehi Jouzani

**Affiliations:** 1 Department of Plant Protection, College of Agriculture and Natural Resources, University of Tehran, P.O. Box 4111, Karaj, Iran; 2 Agroscope, Grazing Systems, Route de la Tioleyre 4, 1725 Posieux, Switzerland; 3 Microbial Biotechnology Department, Agricultural Research, Education and Extension Organization (AREEO), Agricultural Biotechnology Research Institute of Iran (ABRII), Fahmideh Blvd, P.O. Box: 31535-1897, Karaj, Iran

**Keywords:** *Azospirillum oryzae*, *Bacillus velezensis*, Co-culture, Enzyme activity, Swarming, Swimming, Surfactin

## Abstract

The objective of the present study was to evaluate possible interactions between two potential plant growth-promoting bacteria (PGPB): *Azospirillum oryzae* strain NBT506 and *Bacillus velezensis* strain UTB96. To do this, the growth kinetic, biofilm formation, motility, surfactin production, indole-3-acetic acid (IAA) production, phosphate solubilization and enzyme activities of the strains were measured in monoculture and co-culture. The maximum biomass production for the strains in monoculture and co-culture was about 10^11^ CFU/ml, confirming that these two strains have the potential to grow in co-culture without reduction of biomass efficiency. The co-culture system showed more stable biofilm formation until the end of day 3. *Azospirillum* showed the maximum IAA production (41.5 mg/l) in a monoculture compared to other treatments. Surfactin promoted both swimming and swarming motility in all treatments. The *Bacillus* strain in the monoculture and co-culture showed high phosphate solubilizing capability, which increased continuously in the co-culture system after 6 days. The strains showed protease, amylase and cellulase activities in both monoculture and co-culture forms. Chitinase and lipase activities were observed in both the monoculture of the *Bacillus* strain and the co-culture. Overall, our findings highlight the promotion of biological and beneficial effects of these bacteria when growing together in co-culture.

**Supplementary information:**

The online version contains supplementary material available at 10.1007/s11274-022-03283-8.

## Introduction

Co-inoculation of different plant growth-promoting rhizobacteria (PGPR) and biocontrol agents (plant probiotics) has recently been considered as an innovative approach in plant protection and nutrition management, as well as to enhance crop yield and quality (del Orozco-Mosqueda et al. 2018). Indeed, the application of a single plant probiotic strain as inoculant has often shown non-consistent efficiency in the field (Felici et al. 2008). One of the hypotheses of such failure could be the lack of survival, adaptation and /or activity of a single PGPR in all soils due to contrasted biotic and abiotic environmental conditions. An approach to prevail this issue is to use different plant probiotic strains (i.e., microbial cocktails) in a unique formulation (Marimuthu et al. 2002). Application of two or more plant probiotics would simulate the natural flora more closely and might increase the range of biocontrol or PGPB activities (Nikolić et al. 2019). In both agricultural and forest systems, the combination of two or more bacteria or fungal species often induced better results in improving plant growth than application of a single species or strain. While it has been well demonstrated that microbial interactions are playing significant roles in sustainable agriculture by supplying different nutrients or protecting plants from various diseases, the selection and use of suitable and efficient combinations of different microbial species and strains remain challenging (Rojas et al. 2001).

The gram-positive bacteria from *Bacillus* genus, especially strains of *B. velezensis* have been widely used as plant probiotics worldwide. These species have been proved to be effective biocontrol agents against different plant pathogens, such as bacterial wilt, *Fusarium* wilt, *Rhizoctonia* root rot, *Pythium* root rot, and other diseases (Rabbee et al. 2019). Strain UTB96, isolated from pistachio nuts in 2009, has significant biocontrol effects against plant pathogens and reduce aflatoxin (Bagheri et al. 2018). It was originally identified as *B. subtilis* UTB96 but was later reclassified as *B. amyloliquefaciens* UTB96 and registered in GenBank with accession number KY992857 (Bagheri et al. 2018) and is introduced as *B. velezensis* UTB96 based on 16s rDNA analysis in this study and whole genome sequence in the research of Vahidinasab et al. (2019). Commonly, the known biocontrol mechanisms of these species include competition for nutrients and space with other species in the rhizosphere, induced resistance, inactivation of the pathogen’s enzymes, and production of different metabolites, such as antibiotics, siderophores, enzymes and hydrogen cyanid (Khan et al. 2018). Moreover, the production of heat and drought resistant spores makes *Bacillus* spp. a suitable commercialized plant probiotic, as it can be readily formulated into stable products to protect from environmental stresses (Pérez-García et al. 2011).

*Azospirillum* is another soil bacterium from the alpha subdivision of Proteobacteria, which colonizes the roots of numerous economically key crops, such as wheat, rice and corn, and enhances their growth. *Azospirillum* spp. are diazotrophs that fix nitrogen, and some of them show many plant advantageous qualities; such as phytohormones production like auxins, cytokinins, gibberellins, and other plant growth regulators, such as abscisic acid and nitric oxide, ACC deaminase activity, associative nitrogen fixation and control of parasitic plants and bacteria (Combes-Meynet et al. 2011; Puente et al. 2019). The *Azospirillum* strain NBT506 is an important plant growth enhancer that is used as formulation products in the field (Biorun, Nature Biotechnology Company, Iran). *Azospirillum* is however not recognized as a characteristic biocontrol agent of soilborn plant pathogens since lots of strains lack the direct suppressive chemicals effects or the hydrolytic enzymes that disrupt plant pathogens development. Some probable mechanisms used by *Azospirillum* to decrease pathogens infestation have been confirmed as environmental competition and displacement of pathogens, inhibition of seed germination of parasitic weeds, general improvement of plants to naturally fight pathogen contamination and potential inhibition of fungal growth through production of microbial toxic substances (Bashan et al. 2011).

Co-culture of microorganisms with different metabolic capacities (N_2_ fixation, P utilization, production of phytohormones, and antimicrobials, etc.) could affect additive or synergistic effects of different phyto-beneficial capabilities, which might be essential to increase plant protection against pathogens. Some studies have focused on *Azospirillum* double inoculation with other *Azospirillum* (Bashan et al. 2000), *Bacillus* (El-Komy 2005), phosphate-solubilizing bacteria (Belimov et al. 1995), *Pseudomonas* (Combes-Meynet et al. 2011) strains, but only some of the combinations have highlighted improved plant growth compared to the single inoculation. Depending on the strain mixture, microbial interactions inside these associations had positive or negative effects regarding inoculant establishment on roots and occasioned or not enhanced plant growth compared to single inoculation (Couillerot et al. 2013).

The purpose of this study was to assess the optimal conditions (temperature, duration and media) for the growth of two prospective PGPBs, *Azospirillum oryzae* NBT506 and *Bacillus velezensis* UTB96, growing in a co-culture system. *(A) oryzae* is a well known plant growth promoter because of its phytohormones, especially indole acetic acid (IAA) production (i.e., the main property of rhizosphere bacteria that stimulate and facilitate plant growth), whereas *(B) velezensis* is famous for its biological control of pathogens (Bagheri et al. 2018). Combining both bacteria might thus produce suitable conditions for both plant growth enhancement and pathogen suppression. Thus, we aimed to compare the effects of both strains growing in monoculture and co-culture on enzyme activity, biofilm formation, IAA, phosphate-solubilization and bacterial motility, which are important factors in plant-growth promotion. To our knowledge, this is the first report of the interactions between *(A) oryzae* and *(B) velezensis*, aiming to evaluate behavior and biological functions of such strains in both monoculture and co-culture systems. Ultimately, the goal of this study was also to highlight synergistic effects of the two strains for enzymes and surfactin production that could be used to develop new metabolomics assay and formulation products.

## Materials and methods

### Bacterial strains and growth conditions

*Bacillus velezensis* strain UTB96 was obtained from a bacterial collection of University of Tehran, Iran. The *Azospirillum oryzae* strain NBT506 was obtained from Nature biotechnology Company (Biorun, Karaj, Iran). Main media for *Azospirillum* spp. is based on the use of N-free semi-solid media (Nfb), containing low concentrations of agar and a nutrient-rich *Potato* medium (Reis et al. 2015). The *Azospirillum* minimal liquid medium (OAB) which likewise covers high phosphate levels, was prepared and used according to Okon et al. (1977). In addition, the Congo Red agar medium was used according to Cassan et al. (2015). This medium permits the recognition and purification of *Azospirillum* colonies on the plates (Bashan and De-Bashan 2010). Some general commercial microbiological media, such as Luria broth (LB), nutrient broth (NB) and tryptic soy broth (TSB) that are beneficial for experimental laboratory propagation of both *Azospirillum* spp. and *Bacillus* spp. were used in this study as well.

### Molecular identification of the bacteria

Genomic DNA of *Azospirillum* and *Bacillus* was isolated using the CTAB method (Green et al. 2012). The *16 S rDNA* gene fragment was amplified by the general primers PAF (AGAGTTTGATCCTGGCTCAG) and PAR (AAGGAGGTGATCCAGCCGCA) (Zakaria et al. 2010). Each PCR reaction mixture (25 µL) contained 8 µL of PCR master mix, 1 µL of each 10 µM forward and reverse primers, 2 µL of bacterial DNA and 13 µL distilled water. The reaction mixture was incubated in a thermocycler (Eppendorf, Germany) at 94 °C for 3 min, followed by 38 cycles of 94 °C for 40 s, 56 °C for 40 s, and 72 °C for 1 min, and a final extension at 72 °C for 10 min. An amplified PCR product with 1500 bp sizes was separated on 1% agarose gel electrophoresis and stained with ethidium bromide. The PCR products were purified and sequenced by Bioneer Company (South Korea). The gene sequences were trimmed by BioEdit software and compared to sequences in the GenBank database by NCBI and EzTaxon. Sequence alignment and study of gene similarity were carried out using the Clustal W, evolutionary distances were designed, and phylogenetic trees were built with the Maximum likelihood (ML) process. The topology of the trees was assessed by bootstrapping with 1000 resamplings. Phylogenetic trees were drawn with the MEGA6 program (Hall and Krieg 1983). *E. coli* K-12 and *Pseudomonas* spp. were taken as outgroup.

### Kinetics of bacterial growth

The strains were cultured overnight in erlenmyer flasks with LB medium at 24 °C and the bacterial population was 10^10^ CFU. Populations of each strain were adjusted to 10^8^ CFU/L with sterilized distilled water (optical density was measured by spectrophotometry). Population of both strains were diluted 1:100, re-grown in LB medium in a shaker incubator at 37 °C and 160 RPM to count the CFU and read the optical density (OD) at 600 nm at the same time for monoculture and co-cultures of the strains every two hours. We used an incubation temperature of 37 °C when preparing precultures of both bacteria as previous tests with temperatures of 28, 30, 32, 34 and 37 °C showed that the latter was the best temperature for fast bacterial growth. To prevent contamination, separate Erlenmeyers were used every two hours. For CFU counts, cells were diluted serially (10^1^-10^10^) in LB medium and plated on RC agar (ingredients per liter: 0.5 g K_2_HPO_4_, 0.2 g MgSO_4_.7H_2_O, 0.1 g NaCl, 0.5 g yeast extract, 0.015 g FeCl_3_.6H_2_O, 5 g D-Malic acid, 4.8 g KOH, 15 ml CongoRed and 20 g agar) plates with 3 replicates incubated for 24 h at 37 °C. The RC medium was used as a selective medium for *Azospirillum* which appears in red. Mix culture was thus clearer in the RC compared to other media, meaning that both bacteria can differentiate on RC medium. The OD of both strains was measured at the same time every two hours for 48 h using a spectrophotometer (T70 + UV/VIS spectrometer PG Instruments Ltd). Counting of bacteria was done using total viable plate count method, and the number of bacteria was then counted using colony counter and CFU software (http://opencfu.sourceforge.net/). To optimize pre-culture concentration for each strain in the co-culture system, equal biomass of each strain (10^8^ CFU/ml) was used at the first step. Then, different concentrations of each strain were evaluated (10^5, 6, 7^ CFU/ml of NBT506 and 10^4^ CFU/ml of UTB96, 10^8, 9, 10^ CFU/ml of NBT506 and 10^8, 6^ CFU/ml of UTB96) in order to find the best co-culture of both bacteria.

### Biofilm formation

Biofilm formation was calculated as previously described by O’Toole et al. (2000) using a multiwall microtiter plate. The UTB96 and NBT506 strains (100 µl in co-culture and 200 µl in monoculture) with different concentrations and different media were added to the well and were allowed to develop biofilm at 24 °C (without agitation) for three different duration: 24 h, 48 and 72 h. The fresh medium without bacterial cells in the well served as a control and the experiment was replicated three times. To avoid evaporation, the plates were closed with their lid and sealed with parafilm. To evaluate biofilm formation, each well was washed three times using PBS buffer (phosphate-buffered saline) under sterile conditions to eliminate unbound bacteria. The crystal violet 0.1% (v: v) was used to monitor the wells, and incubated for 30 min at room temperature. Then, plates were washed carefully three times with tap water to remove extra crystal violet. Dye attached to the wells was extracted with 200 µL of 33% (v: v) acetic acid in water. The absorbance was determined at 492, 590, 540 and 600 nm using a microplate reader (O’Toole 2011). Total cell number of planktonic and biofilm-forming bacteria was estimated by measuring the OD at 540 or 600 nm. Adhesion rate was calculated as the proportion of biofilm-forming bacteria on total bacteria: OD 590 nm / OD 540 nm (Salcedo et al. 2015).

### Effect of *Azospirillumoryzae* on surfactin production in *Bacillusvelezensis*

The effect of the *A. oryzae* strain (NBT506) cell and cell-free cultures on surfactin production of the *B. velezensis* strain (UTB96) was measured. To do this, 1% of 10^8^ CFU/ml of NBT506 population and 10^6^ CFU/ml of UTB96 population inoculated to LB medium in monoculture and co-culture, and NBT506 cell free culture added 50% (v/v) to LB medium (25 ml cell-free culture + 25 ml LB) for 48 h in 37 °C. Then, biosurfactant was extracted and quantified according to the method previously designated by Liu et al. (Liu et al. 2008). Bacterial cells in LB were removed from the culture by centrifugation at 8000–10,000 rpm for 20 min at 4 °C. The supernatant was subject to acid precipitation by the addition of 6 M HCl to a final pH of 2 and allowing the precipitate to settle overnight at 4 °C. The precipitant was collected by centrifugation at 8000–10,000 rpm for 20 min at 4 °C. To extract biosurfactant compounds, chloroform was added to the pellet and incubated in a rotatory shaker at 250 rpm, 30 °C (± 0.5 °C) for 15 min. Then pellets were lyophilized for quantification. Assesses were approved in triplicates. The quantification of surfactin was carried out using a Waters (Milford, USA) HPLC equipped with an X-Terra reverse phase C18 column according to the method previously described by Wei and Chu (Wei and Chu 2002). Commercial surfactin (Sigma–Aldrich, St. Louis, USA) was used as a standard.

### Bacterial motility

The soft agar plate assay was performed using a low concentration of agar to allow cells swimming with the polar flagellum and swarming. The cells from an overnight culture were inoculated in the center of a soft agar plate (90 mm) having 0.2% agar for swimming (moving into liquid media) and 0.7% agar for swarming (moving in a semi-solid media) assay in 0.1 TSBA media. The treatments included surfactin, biosurfactant of the UTB96 strain, the UTB96 strain cells (10^6^ CFU/ml) and the UTB96 strain cell-free culture that were added in an amount of 100 µl in each plate, followed by the inoculation of 5 µl of the NBT506 strain (10^8^ CFU/ml) culture inoculated in the center of each plate after solidification of media. Other treatments included surfactin, biosurfactant of the NBT506 strain, the NBT506 strain cells (10^8^ CFU/ml), and the NBT506 cell-free culture that were added in an amount of 100 µl in each plate as described before, followed by the inoulcation of 5 µl of the UTB96 strain cells (10^6^ CFU/ml) in the center of each plate after solidification of the media for 24 h at 24 °C. Measurement of the halo diameter highlighted movement activity. The NBT506 and UTB96 strains in 0.1 TSBA media with 0.2% and 0.7% agar were used as control.

### IAA production

Determination of indole-3-acetic acid (IAA) was performed by spectrophotometry according to Salkowski (1885). The NBT506 and UTB96 strains were cultured overnight in LB or TSB media, then after determining their concentration, new 0.1 TSB media with 5 mM tryptophan were inoculated in triplicate with 1% of 10^8^ CFU/ml of the NBT506 and 10^6^ CFU/ml of the UTB96 in monoculture and co-culture. Also, to determine the effect of commercial surfactin in the IAA production of NBT506, it was added with 0.1 mg/ml concentration to a monoculture of 1% of 10^8^ CFU/ml of the NBT506 strain. The flasks were incubated for 24, 48 and 72 h at 24 °C with 140 rpm orbital agitation. Uninoculated flasks were used as control. One ml aliquot of each flask was collected 24, 48, and 72 h after inoculation and centrifuged at 8000 rpm for 10 min at 4 °C, then the supernatant was mixed strongly with 4 ml of Salkowski’s reagent and allowed to stand in the dark at room temperature for 30 min. Thereafter, production of IAA in the culture supernatant was measured by determining the absorbance at 535 nm wavelength using a spectrophotometer.

### Qualitative assessment of phosphate-solubilization by the strains in PVK medium

Phosphate solubilizing was assayed using the method previously described by Pikovskaya (1948). PVK medium was supplemented with 1.7% agar and the pH was adjusted to 7.0 before autoclaving. The strains were stabbed in triplicate per plate using sterile toothpicks. The halo and colony diameters were measured 2, 4 and 6 days after the incubation of plates at 24 °C. Halo size was calculated by the following formula: SE = Solubilization diameter / Growth diameter × 100.

### Determination of enzyme activities

The activities of five enzymes, including lipase, protease, amylase, cellulase and chitinase were studied based on the agar plate methods. The concentrations of enzymatic hydrolysis products were determined by halo zone after 48 h of incubation at 24 °C. The protease activity was determined in SMA media according to Maurhofer et al. (1995). Five µl of each bacterium in mono and co-culture systems were incubated in the center of the plates. The lipase activity was assayed on the plates containing Tween 80% (Schaad et al. 2001). For cellulase activity, 5 µl of each bacterium was spot plated on CMC agar (Gohel et al. 2014). Chitinase production was performed by culturing bacterial cultures on the colloidal chitin agar medium (Kuddus and Ahmad 2013). The bacterial cultures were screened for amylolytic activity using the starch hydrolysis test on starch agar plates (Shaw et al. 1995). Grams iodine stain were used as the plate assay method for determining cellulase, amylase and chitinase activity as it gives the best distinct clear zones within 2–3 min and is not toxic for the cells (Gohel et al. 2014).

### Statistical analysis

All statistical analyses were carried out in the R environment (version 4.0.2). The treatment effects of the *(A) oryzae* NBT506 and *(B) velezensis* UTB96 strains on optical density and Log CFU in monoculture and co-culture systems were tested separately for each time (every 2 h from 0 to 24 h) using ANOVAs followed by post hoc Tukey tests to highlight the significant difference between strains and culture system (monoculture and co-culture). The effects of different media on biofilm formation of the *(A) oryzae* NBT506, as well as the effect of different strains + metabolites on swimming and swarming, were tested using ANOVAs followed by post hoc Tukey tests. The interactive effects of bacterial strains + metabolites combinations (*A. oryzae* or *(B) velezensis* + CFC, BS or surfactin) and time (24, 48 or 72 h) on biofilm formation (optical density at 590 nm) and IAA production were tested using ANOVAs, specifying ‘time’ as error term for repeated measures, followed by post hoc Tukey tests. The effects of *(A) oryzae* NBT506 and *(B) velezensis* UTB96 (monoculture and co-culture) on phosphate solubilization after 2, 4 and 6 days, were tested using ANOVAs specifying ‘time’ as error term for repeated measures, followed by post hoc Tukey tests.

## Results

### Bacterial identification

**Fig. 1:**
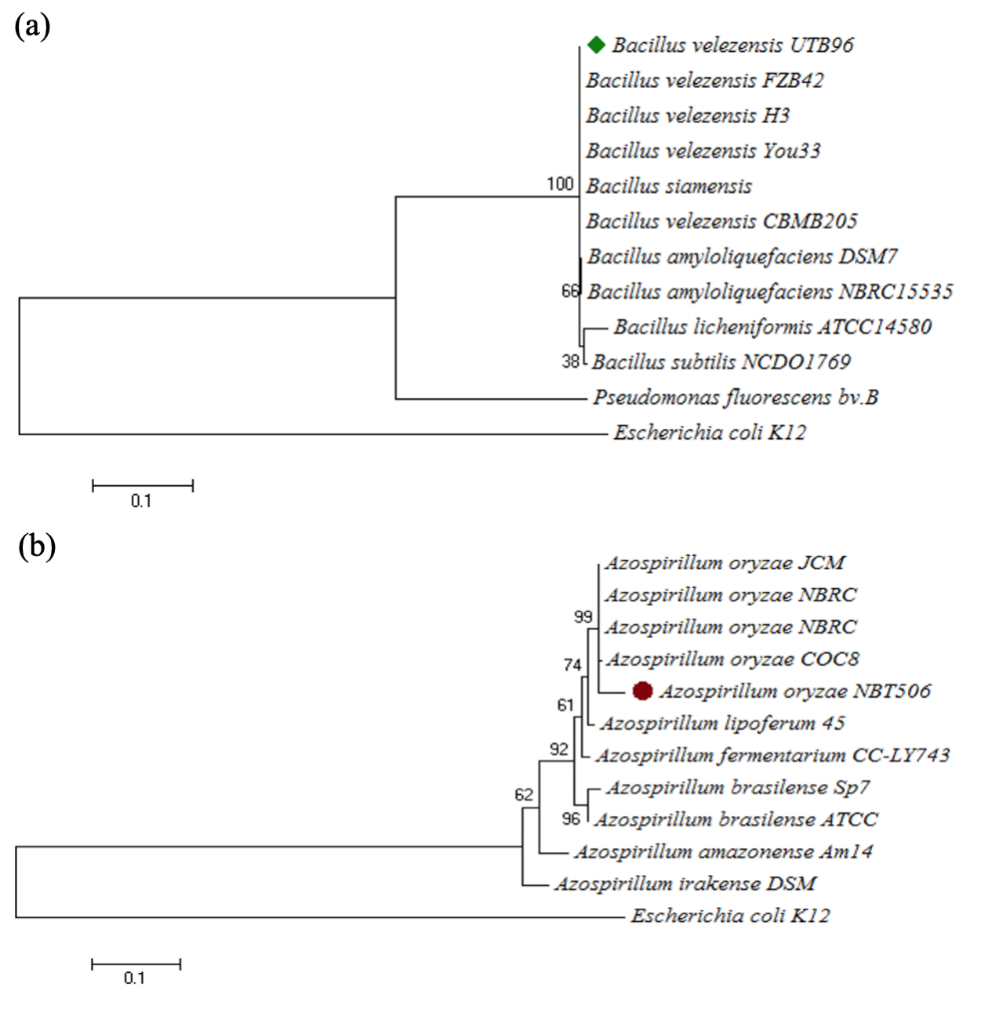
Phylogenetic tree of (a) *Bacillus velezensis* strain UTB96 and (b) *Azospirillum oryzae* NBT506, based on *16 S rRNA* gene sequences

The amplification (1500 bp) and sequencing of the *16SrRNA* gene from both *Bacillus* and *Azospirillum* strains showed that the strains have more than 99% similarity with *B. velezensis* and *(A) oryzae* species, respectively. The sequences were submitted to GenBank EMBL with name *(B) velezensis* UTB96 and *(A) oryzae* NBT506. The results of phylogenetic trees are presented in Fig. 1. Based on this whole-genome sequencing research, *(B) velezensis* UTB96 was submitted to NCBI with accession number NZ_CP036527, as previously described in Vahidinasab et al. (2019), and *A. oryzae* NBT506 was submitted with accession number MH973635.

### Bacterial growth kinetics

**Fig. 2:**
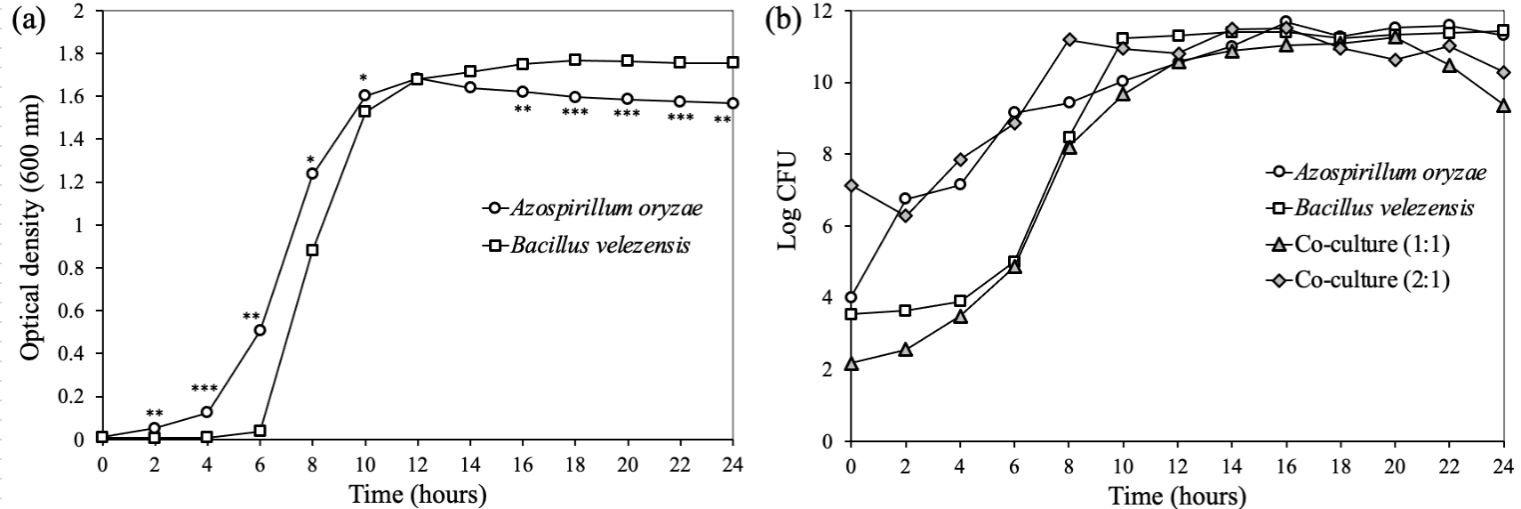
Growth kinetics of *Azospirillum oryzae* NBT506 and *Bacillus velezensis* UTB96 in monoculture and co-culture systems. (a) Optical density at 600 nm of monocultures of *Azospirillum oryzae* NBT506 (open circle) and *Bacillus velezensis* UTB96 (open square) during 24 h. Stars indicate significant difference between both species at each time (* P < 0.05; ** P < 0.01; *** P < 0.001). (b) Log CFU (CFU.ml^− 1^) of monocultures of *Azospirillum oryzae* NBT506 (open circle), monoculture of *Bacillus velezensis* UTB96 (open square), co-culture of the same population size of each strain (10^8^ CFU/ml; grey triangle) and co-culture when NBT506 (10^10^ CFU/ml) population is two-fold more than UTB96 (10^8^ CFU/ml) during 24 h. In (a) and (b), standard errors were too small to be visible on the graph and have thus been omitted

The results showed that the strain *B. velezensis* UTB96 reached its maximum growth after 24 h with 3 × 10^11^ CFU/ml, whereas the strain *A. oryzae* NBT506 reached its maximum growth after 16 h with 4 × 10^11^ CFU/ml in the monoculture systems (Fig. 2). The bacterial growth changed in the co-culture of both species when the same population of each strain (10^8^ CFU/ml) and the amount of 1% of preculture was used, in this situation the NBT506 strain biomass and average biomass of co-culture were decreased. So different preculture concentrations were used for both strains, results showed that when the NBT506 population as preculture is a fold of 2-logs more than that of the UTB96 strain, maximum biomass for both strains was achieved (Table 1). Application of 1% of 10^8^ CFU/ml of the NBT506 strain and 10^6^ CFU/ml of the UTB96 strain showed maximum growth efficiency for both strains (Fig. 2). This showed that in co-culture of the NBT506 and UTB96 strains, both bacterial biomasses were active and at the same time biomass of both bacteria were increased in the co-culture system at the early stage of growth. Such findings are extremely useful to create multi-microbial formulations using both strains. In this case, some benefits will observe in an economical matter such as using less medium with more efficiency.

**Table 1:**
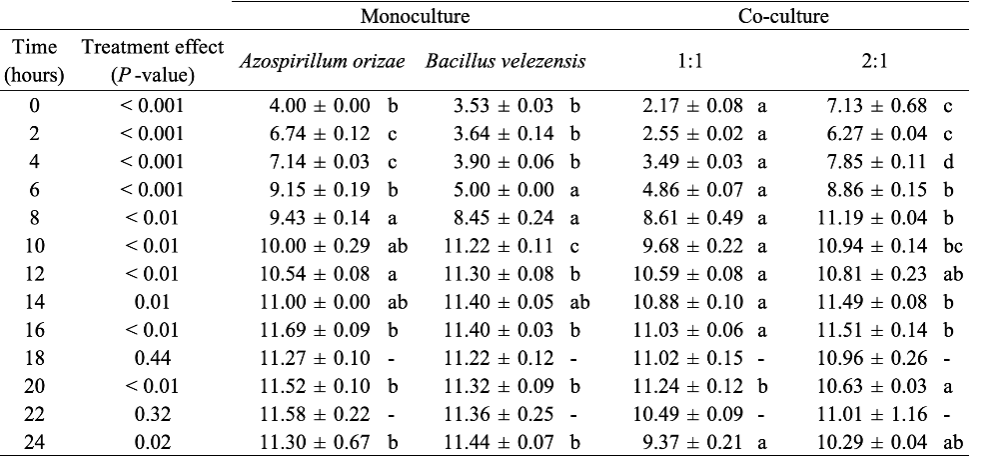
Statistical outputs of the ANOVAs testing the effects of *Azospirillum oryzae* NBT506 and *Bacillus velezensis* UTB96 strains in monocultures and co-cultures (1:1 same population size of each stain; 2:1 two-fold more NBT506 compared to UTB96) on Log CFU (CFU.ml^− 1^) along the time (as represented on Fig. 2b). Significant differences among monocultures and co-cultures at each time (Anovas) are indicated by different letters

### Biofilm formation assay

**Fig. 3:**
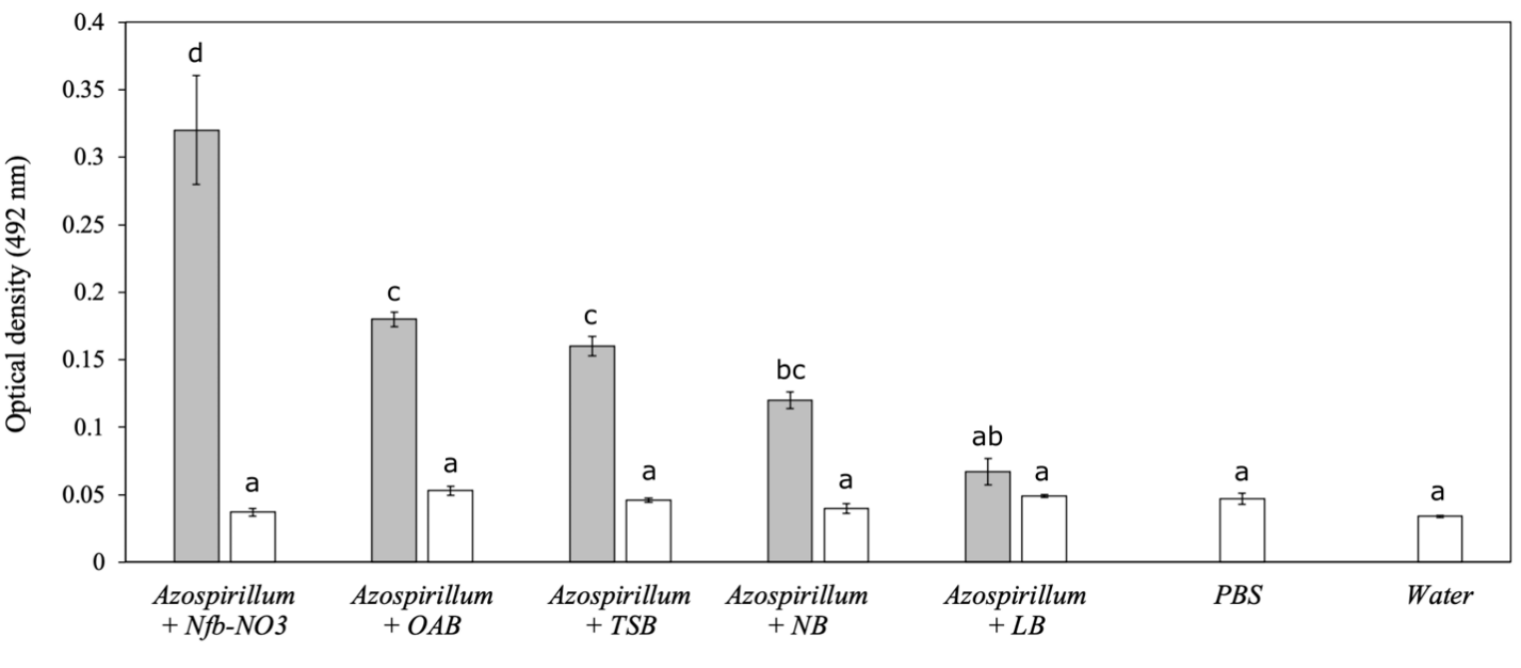
Biofilm formation (i.e., optical density at 492 nm ± SE) of the *Azospirillum oryzae* NBT506 strain in different media (each media is also applied as a control without bacteria and represented with white bars). Different letters highlight significant differences among media (*P* < 0.05). Azospirillum *= Azospirillum oryzae* Nfb-NO3 = N-free semi-solid media with nitrate ; OAB = minimal liquid medium ; TSB = tryptic soy broth ; NB = nutrient broth; LB = Luria broth. ; PBS = phosphate-buffered saline

The NBT506 strain showed significantly different amounts of biofilm in different media (Fig. 3). The maximum biofilm formation for this strain was observed in Nfb-NO_3_, OAB and TSB media, respectively, whereas the minimum of that observed in the NB and LB media (Fig. 3). A different combination of the studied strains and metabolites (NBT506, UTB96, co-culture, biosurfactant (BS) and cell-free culture (CFC) of each strain and surfactin) showed the significantly different capability of biofilm formation in LB medium during 3 days (*P* < 0.01). After 24 h, the UTB96 + NBT506 biosurfactant, following by the UTB96 strain showed the highest biofilm formation. This means that the NBT506 biosurfactant plays a major role in influencing the UTB96 biofilms formation in the early stage. The second highest biofilm formation belonged to UTB96 + surfactin and NBT506 + UTB96 cell-free culture. After 48 h, UTB96, UTB96 + NBT506 biosurfactant, and NBT506 + UTB96 cell-free culture showed highest amounts of biofilm formation with significant differences (*P* < 0.01). Then data was normalized by total growth estimated to evaluate the number of cells in the biofilm comparative to total culture growth. We observed that the UTB96 + NBT506 cell-free culture, the UTB96 + surfactin, the NBT506 and co-culture had a significantly higher biofilm formation (*P* < 0.01). After 72 h, the NBT506 + UTB96 cell-free culture and NBT506 treatments showed the highest biofilm formation among all treatments. These two treatments formed more stable biofilms, which showed high stability after 3 days. Finally, it could be concluded that the maximum biofilm formation has occurred after 48 h, and after that, the biofilm contents continuously decreased.

**Fig. 4:**
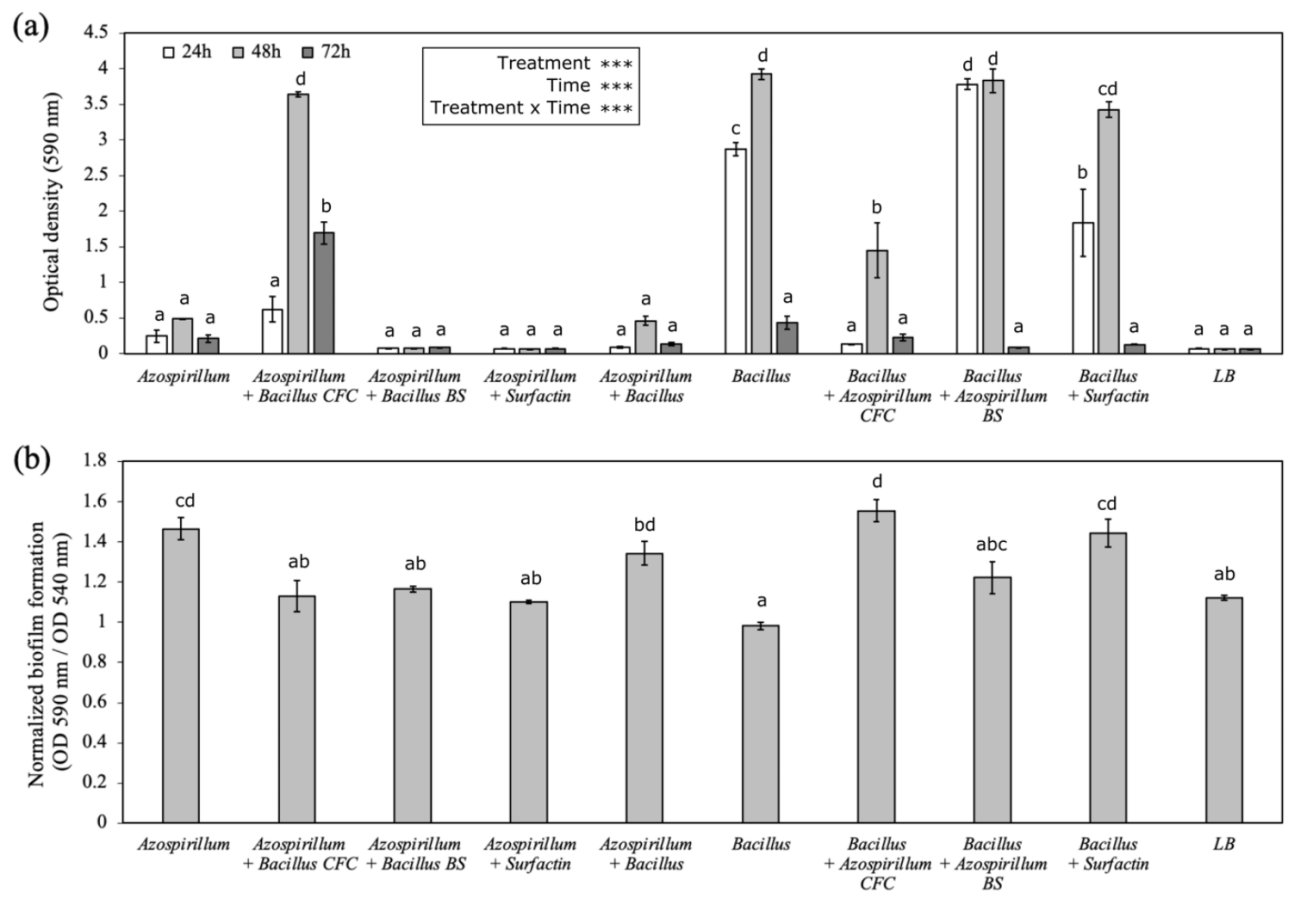
Biofilm formation of different strains and metabolites combinations (i.e., treatment). (a) Optical density (OD 590 nm ± SE) after 24 h, 48 and 72 h (i.e., time); (b) Normalized biofilm formation (OD 590 nm / OD 540 nm ± SE) after 48 h. *Azospirillum* = *Azospirillum oryzae* NBT506; *Bacillus* = *Bacillus velezensis* UTB96; CFC = cell-free culture; BS = biosurfactant; LB = Luria broth. Stars indicate significant effect of the treatments, time and their interactions (*** P < 0.001) and different letters highlight significant differences among treatments (*P* < 0.05)

In the co-culture treatment, the biofilm formation was significantly (*P* < 0.01) lower than the monoculture of the UTB96. Our results also showed that commercial surfactin not only did not increase biofilm formation in NBT506 but also reduced the biofilm contents compared to the NBT506 monoculture (Fig. 4). Biofilm formation in the co-culture system occured later than the monocultures, but biofilm formation was more stable in this system and continued till the end of day 3. The maximum biofilm formation was observed after 48 h for the treatments UTB96, UTB96 + NBT506 biosurfactant, and NBT506 + UTB96 cell-free culture.

### HPLC analysis of surfactin production

The retention times for surfactin were recorded at 8.15, 9.13, 15.47, 26.7, 28.8, 35.11, 38.13, 45.5 and 46.6 min. The calibration curve graph for surfactin was constructed by plotting the total peak area against various concentrations of surfactin standard. The HPLC analysis showed that when the NBT506 cell-free culture was added to the UTB96 strain and co-culture, more surfactin was produced compared to the monoculture of the UTB96 strain (270, 260 and 220 mg/l, respectively).

### Bacterial motility

**Fig. 5:**
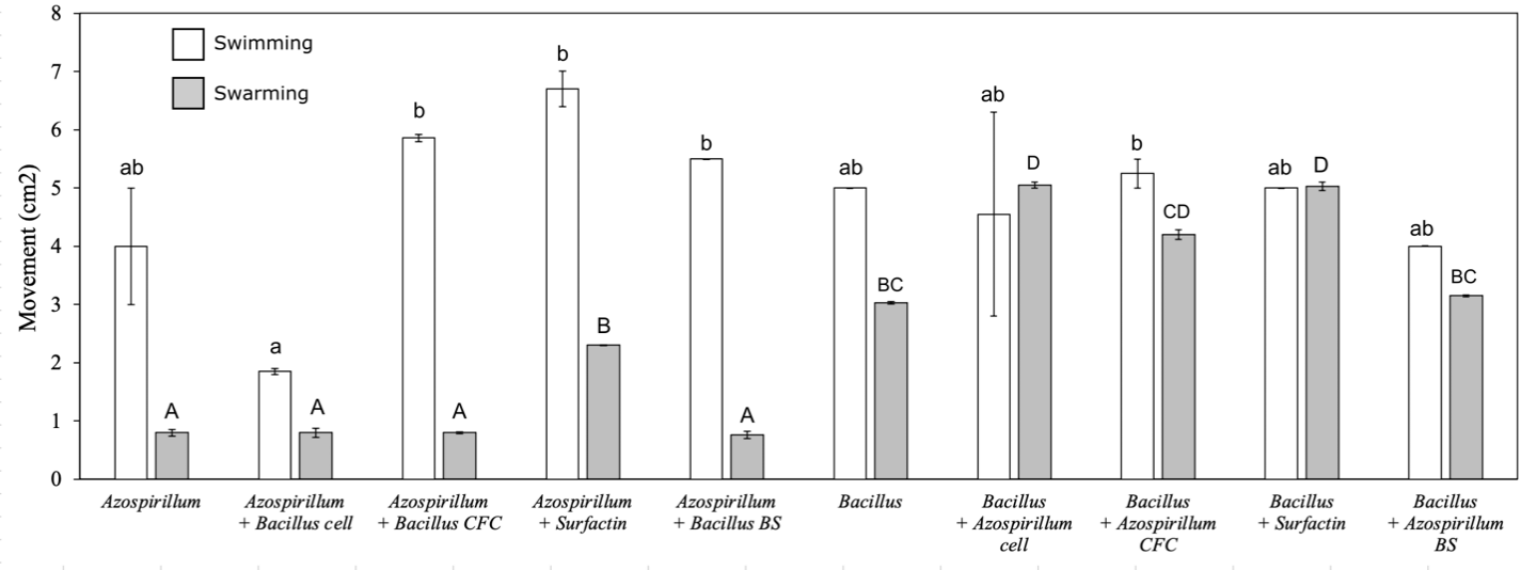
Swimming (white bars) and swarming (grey bars) of different strains and metabolites combinations determine through movement (cm^2^). *Azospirillum* = *Azospirillum oryzae* NBT506; *Bacillus* = *Bacillus velezensis* UTB96; CFC = cell-free culture; BS = biosurfactant. Different letters (i.e., lower-case letter for swimming and capital letters for swarming) highlight significant differences among treatments (*P* < 0.05)

The motility assays highlighted different motility behaviors of the two studied bacteria in the different treatments, especially during the swimming stage. The swimming movements of UTB96 was not significantly different from those of NBT506 in the monocultures without additives (Fig. 5). When the NBT506 was inoculated in the middle of plates with different treatments, TSBA medium with surfactin and UTB96 cell-free culture showed significantly (*P* < 0.05) more motility compared to the control (NBT506 in the center of the TSBA medium without any additive), respectively. However, for UTB96, application of the TSBA medium with NBT506 cell-free culture and surfactin did not influence motility compared to the control (i.e., UTB96 in TSBA medium). Therefore, the cell-free culture of UTB96 bacterium could stimulate swimming movement in NBT506 bacteria, whereas surfactin can stimulate swimming movement for the NBT506 strain.

The results of swarming assays showed that application of surfactin in the medium could significantly increase motility of the NBT506 strain (*P* < 0.05), and this treatment had maximum motility among all treatments (Fig. 5). When the NBT506 cells and surfactin were used as an additive in the plates of The UTB96, maximum swarming were observed respectively, compared to the control (the UTB96 strain in the center of the TSBA medium without any additive) (*P* < 0.05). Comparison of the NBT506 and UTB96 strains in the monoculture showed that the UTB96 strain was significantly more mobile in the swarming stage than the NBT506 (*P* < 0.05), and that the use of surfactin could promote both swimming and swarming motility in the treatments (Fig. 5).

### IAA production

**Fig. 6:**
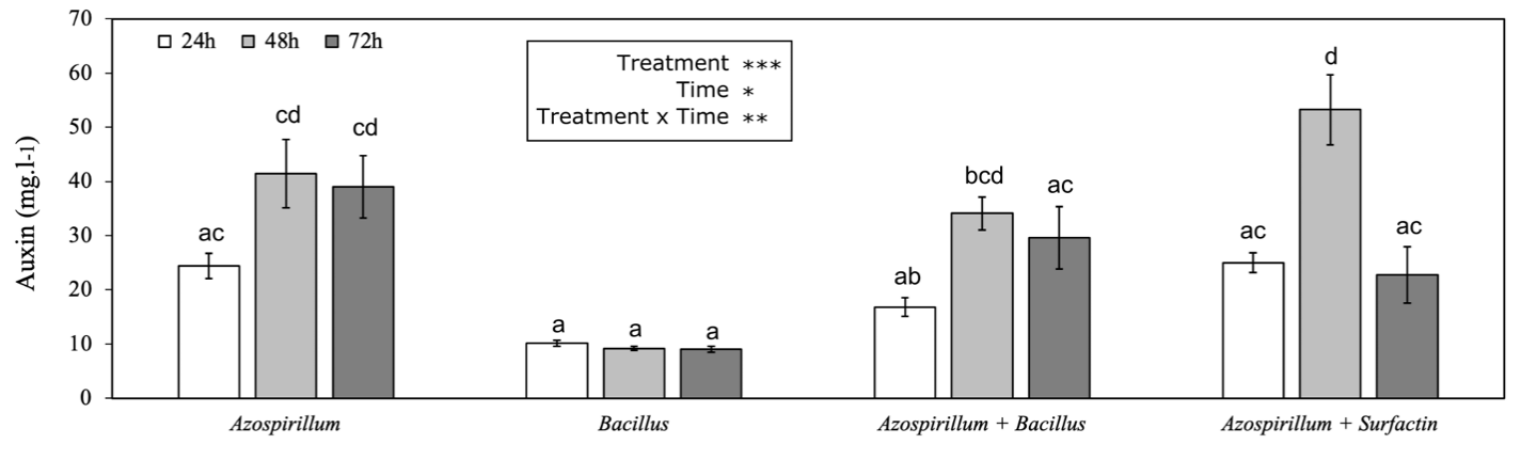
Indole-3-acetic acid (IAA) production (mg.l^− 1^) by the different treatments: monoculture of *Azospirillum oryzae* NBT506 without and with surfactin, monoculture of *Bacillus velezensis* UTB96 strains, co-culture of *Azospirillum oryzae* NBT506 and *Bacillus velezensis* UTB96, and LB media (shown as reference) after 24 h, 48 and 72 h. Stars indicate significant effect of the treatments, time and their interactions (*** P < 0.001) and different letters highlight significant differences among treatments (*P* < 0.05)

Different treatments produced significantly different amounts of auxin (*P* = 0.01) (Fig. 6). The monoculture of the NBT506 strain without any additives showed the highest IAA production during the 3 days (24.38, 41.47 and 39.06 mg/l, respectively at 24, 48 and 72 h) among all treatments by comparison to UTB96 (10.16, 9.17 and 9.03 mg/l, respectively at 24, 48 and 72 h). In addition to a monoculture of NBT506, NBT506 with surfactin and co-culture of NBT506 and UTB96 showed higher amounts of auxin. Moreover, the IAA production reached its maximum level after 48 h for all treatments. The NBT506 strain showed a stable auxin production after 48 h in both mono and co-culture systems (Fig. 6).

### Phosphate-solubilization

**Fig. 7:**
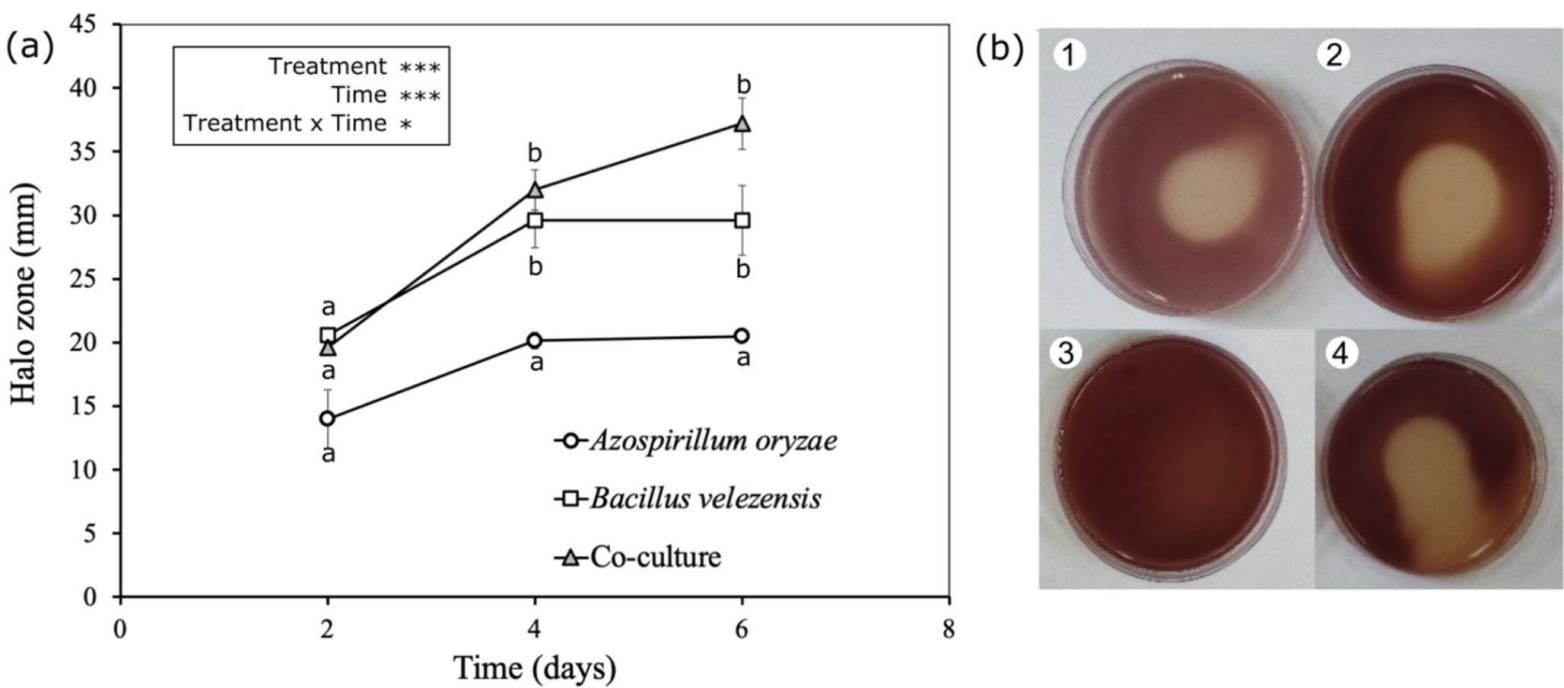
(a) Phosphate solubilization (halo zone in mm) of the *Azospirillum oryzae* NBT506 and *Bacillus velezensis* UTB96 strains in the monoculture and co-culture. (b) Halo zone in PVK plate after 4 days: 1: *Azospirillum oryzae* NBT506, 2: *Bacillus velezensis* UTB96, 3: control, and 4: co-culture of *Bacillus velezensis* and *Azospirillum oryzae.* Stars indicate significant effect of the treatments, time and their interactions (* *P* < 0.05, *** *P* < 0.001) and different letters highlight significant differences among treatments (*P* < 0.05)

The monoculture of the UTB96 strain and also co-culture of the UTB96 and NBT506 strains were the most powerful phosphate solubilizers on Pikovskaya (PVK) plates whereas the monoculture of the NBT506 strain exhibited weak zones of solubilization. The co-culture treatment significantly (*P* = 0.01) enhanced phosphorus solubilization for 6 days (Fig. 7) to compare with NBT506. The UTB96 and NBT506 monoculture solubilization effectiveness increased after 2 and 4 days of incubation, and then the solubilization stopped, whereas the co-culture solubilization efficiency continuously increased after 6 days. Therefore, it could be concluded that the co-culture system keeps UTB96 solubilization efficiency at high levels.

### Enzymes activities assays

The enzyme activities assays, evaluating clearance zones on the media containing coordinated substrates, highlighted that the UTB96 and NBT506 strains showed protease, amylase and cellulase activities in both mono and co-culture formats, and that there was no difference in halo zone diameter among the different treatments (Table 2). The NBT506 strain did not show chitinase and lipase activities, whereas, the UTB96 strain showed these activities in both mono and co-culture.

**Table 2:**
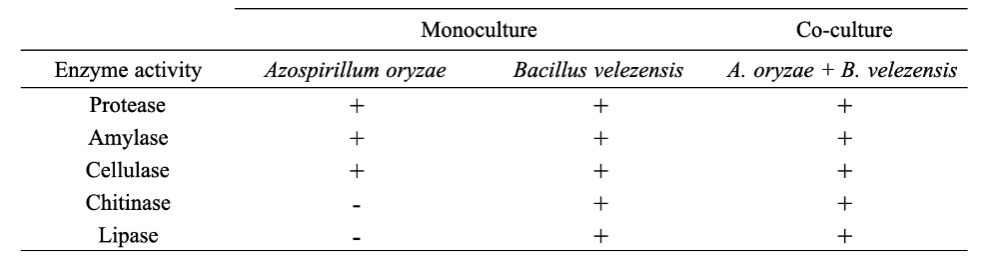
Enzyme activity assays for the monoculture and co-culture of *Azospirillum oryzae* and *Bacillus velezensis.* Presence (positive sign) or absence (negative sign) of protease, amylase, cellulase, chitinase and lipase activities are indicated for each bacterial culture

## Disscussion

The most essential parameters for selecting bacterial strains as PGPR or biocontrol agents are their growth kinetics and biomass production capabilities under various environments. In this study, we compared the importance of mixing *Azospirillum oryzae* NBT506 and *Bacillus velezensis* UTB96 strains in co-culture to monoculture in different growth conditions to see if mixing strains of *Azospirillum oryzae* NBT506 and *Bacillus velezensis* UTB96 improved growth kinetics and biomass production. Our study showed maximum growth of these two strains not only in monoculture but also in co-culture. Application of adjusted population of 10^8^ CFU/ml of the NBT506 strain and 10^6^ CFU/ml of the UTB96 strain showed maximum growth efficiency based on different preculture concentrations for both strains. This population adjustement in co-culture was observed in the research of Masciarelli et al. (2014); *Bradyrhizobium japonicumtiter* was adjusted to 10^9^ CFU/ml, on YEM broth, whereas *B. amyloliquefaciens* was adjusted to 1 × 10^8^ CFU/ml on LB medium. In another study, the mixed population of *Phyllobacterium* sp. and *B. licheniformis* significantly increased during the first 24 h and then constantly decreased (Rojas et al. 2001; Wang et al. 2015) also co-cultured 2 × 10^7^ CFU/ml *Bifidobacterium animalis* with 4 × 10^4^ CFU/ml *B. subtilis* natto to produce *B. animalis* as the main product. On the other research, the population dynamics of *B. subtilis* and *(A) brasilense* had a range of 5.5–6.5 log CFU/g and in co-culture, treatments did not produce significantly different results (Felici et al. 2008). Different preculture biomass of bacteria in co-culture were used in the study of El-Katatny et al. (1997), with pectin or malate containing media with 10^7^ CFU/ml for *Azospirillum* and 4.5 × 10^5^ CFU/ml of *(B) polymyxa*.

Different media based on different nutritional compounds were utilized in the biofilm investigation of *A. oryzae* NBT506, although the Nfb-NO3 medium developed the most biofilm. Salcedo et al. (2015) studied the relationship between compound and biofilm formation in *A. brasilense* Sp245 cells and found that the concentrations of various compounds, such as sucrose, phosphate, and calcium, had a positive correlation with biofilm formation, whereas extreme temperatures and pH values had a negative correlation. Another feature to be taken into the account is the timing of the biofilm formation. Usually, the incubation time can differ from hours to days for different microorganisms and different conditions. As we see in our study the highest limit of biofilm formation was after 48 h. Another survey showed that the adhesion of *Azospirillum* cells in stationary phase is particularly evident 24 h after incubation. The amount of biofilm in individual cultures of Sp245 or CHA0 was significantly lower than the amount of biofilm produced in co-cultures (Pagnussat et al. 2016). Biofilm cause lots of benefits for plant probiotic bacteria such as, protect them from environmental stresses, increase the ability to use nutrition, etc. (Ahmadzadeh 2014).

The NBT506 strain with surfactin and the UTB96 cell-free culture showed significantly more swimming feature. Surfactin secretion could be of biological importance in the life cycle of *B. subtilis*, as it is vital for moving on tissues and also during the process of swarming in *B. subtilis* (Julkowska et al. 2005). Previously, the chemotaxis regulating polar flagellum mediated motility in liquid environments, i.e., swimming, has been well described, but mechanisms involving in swarming motility has yet to be discovered (Alexandre 2015).

Auxin assay of the NBT506 strain monoculture showed the highest IAA production during the 3 days (24.38, 41.47 and 39.06 mg/l, respectively at 24, 48 and 72 h) followed by NBT506 with surfactin and co-culture of NBT506 and UTB96. These values are higher than those found by the study of Felici et al. (2008), which showed that *B. subtilis* 101 and *A. brasilense* Sp245 produce auxin molecules at the rate of 2.60 mg/L and 7.95 mg/L, respectively, after 72 h of incubation in the growth medium without L-TRP. However, the addition of L-TRP to the medium enhanced these values for the strains up to two-fold (5.21 mg/L) and five-fold (40.20 mg/L), respectively. In another study, it was showed that co-inoculation of *Rhizobium* sp. and *Azospirillum* sp. to *Medicago polimorpha* significantly improved the number, weight and nitrogenase activity of root nodules compared to the single-inoculated plants (Yahalom et al. 1990).

Enzyme activity was higher in co-culture in our experiment. The halo size was higher in co-culture for protease, amylase and cellulase activity whereas the monoculture of NBT506 had no lipase and chitinase activity. It has also been observed with other strains in Halsall and Gibson’s study (1985), which showed that co-cultures of *Cellulomonas gelida* with *A. brasilense, A. lipoferum*, or *Bacillus macerans* were able to breakdown cellulose and fix atmospheric nitrogen. Vazquez et al. (2000) showed that co-culture of *Pseudomonas* with *Glomus deserticola* could significantly improve esterase activity in *G. deserticola.* They also confirmed that co-inoculation of *G. deserticola* with either *Azospirillum*, *Pseudomonas* or *Trichoderma* could significantly enhance phosphatase activity in the rhizosphere of *G. deserticola* colonized plants. El-komy (2005) evaluated the ability of phosphate solubilizing in some bacterial species. The results showed that the maximum phosphate solubilization was observed for *Pseudomonas fluorescens* and *Bacillus megaterium* strains whereas *Azospirillum lipoferum* strains displayed weak zones of solubilization on the PVK plates similar to what we found for NBT506. Solubilization efficacy (SE) was constantly increased up to 4 days of incubation, and then the solubilization was decreased and finally stopped (El-Komy 2005).

Overall, our findings highlight that the co-culture of two plant-promoting bacteria, including the strains *(A) oryzae* NBT506 and *(B) velezensis* UTB96, enhanced their biomass production as well as different biological and chemical properties, such as IAA production, motility, phosphate solubilization and enzyme activities. This suggest that this bacterial combination could be more suitable and effective for field applications as plant growth-promoting bacteria than single strain inoculation. Altogether, our results provide new insights and perspectives for the development of better bacterial inoculant for plant growth enhancement and disease suppression. In an additional experiment, assays of the co-culture of *Bacillus velezensis* UTB96 and *Azospirillum oryzae* NBT506 on wheat growth promotion and control of *Fusarium graminearum* already confirmed that these strains prevent mycelium growth of *F. graminearum* by direct inhibition and VOCs and thus improve wheat growth (*see* Bagheri et al. 2019).

## Electronic supplementary material

Below is the link to the electronic supplementary material.

Supplementary Material 1

## Data Availability

The datasets generated during and/or analysed during the current study are available from the corresponding author on reasonable request.
