# Supplementary material for: Behavior and interactions of the plant growth-promoting bacteria Azospirillum oryzae NBT506 and Bacillus velezensis UTB96 in a co-culture system
Source: World J Microbiol Biotechnol. 2022 Apr 29;38(6):101. doi: 10.1007/s11274-022-03283-8 (PMC9054896; doi:10.1007/s11274-022-03283-8)
Supplement: Supplementary file 1 — Supplementary Material 1 [file 11274_2022_3283_MOESM1_ESM.docx]

**AUTHOR’S CHECKLIST BEFORE MANUSCRIPT SUBMISSION**

**A copy of this, appropriately checked and signed, should accompany each submitted paper.**

Your paper must follow the advice given in our **Instructions for Authors** which are available online. Failure to comply with these instructions will delay assessment.

Please **do not submit** your paper if it fits one of the following criteria:

- virological, veterinary, agricultural and clinical topics
- a simple description of an environment or report of a procedure
- report on optimization procedures
- descriptions of the biological properties of undefined extracts or extracts from plants and spices
- microbial culture isolation from local lakes and rivers
- data about not fully purified enzymes
- data reporting the host responses to microbes

Authors whose first language is not English, and those inexperienced in scientific writing, are urged to have their manuscript thoroughly checked before submission. The use of a professional paper writing agency is strongly recommended for those authors who are doubtful of their command of the English language. Papers that are poorly written or are presented in an incorrect style and format cannot be sent out to reviewers. These will be returned to the authors without assessment for revision and rewriting.

**Please do not tick the following boxes until you have complied with the instructions indicated.**

x All authors have read the submitted version of the paper AND have approved its submission.

x Please make sure that you **deposit strains and sequence data** in a recognized data bank

x All authors have double checked their affiliation (with precise location), especially if an email address is used that is not provided by the institution.

x All text, including Abstract, tables, references, footnotes and figure legends, is double-spaced printed

(NOT 1 1/2 spacing) and uses a full-page width (15 cm) on the equivalent of an A4 sheet**.**

x The title should be informative and clear. Do not use unspecified, nonstandard abbreviations in the title. Avoid ambiguous expressions such as “The effect of…”. Please ensure that the biological origin of the system under investigation is given in the title.

x Key words or phrases (but not abbreviations) are given in **alphabetical order** after the Abstract. Up to

7 can be used.

x Do not submit abstracts divided in sections, containing non-standard abbreviations or citing references.

x Sub-headings in all sections should be clearly indicated but are **NOT** numbered.

x Do not combine Results and Discussion.

x Tables and figures are placed after the list of references. (Tables before figures.)

x Legends to figures are grouped together on a separate sheet(s) which precedes the figures themselves.

x Position for each table and figure should be indicated in the text by a marginal note or a clear note

between paragraphs in the text.
